# 

**Published:** 1885-01

**Authors:** 


CONTENTS.

Vol. 32 No. 1

Our New Year's Greeting........ 1

Deep Breathing........ 2

Dangers from Impure Water........ 3

A Miraculous Recovery........ 3

Fashions in Bibles........ 4

Premature Baldness........ 5

Reflex Nervous Influence........ 6

A Fortune for Barbers........ 7

Suppose We Had No Sugar........ 8

Arsenic in Paper........ 10

A New Treatment for Neuralgia........ 11

How Postal Currency Was Invented........ 11

Salt for the Human System........ 12

Poisoning........ 13

Treatment of Cholera........ 13

A New Remedy........ 14

The Color of Water........ 14

A Remarkable Explosion........ 15

Long Rides........ 15

The Unseen Poor........ 16

Notes and Miscellany........ 17-18-19-20

